# Integrated transcriptomic and proteomic analysis of a cytoplasmic male sterility line and associated maintainer line in soybean

**DOI:** 10.3389/fpls.2023.1098125

**Published:** 2023-02-02

**Authors:** Dagang Wang, Yanan Wang, Lei Zhang, Yong Yang, Qian Wu, Guoyu Hu, Weihu Wang, Jiekun Li, Zhiping Huang

**Affiliations:** Key Laboratory of Crop Quality Improvement of Anhui Province, Crop Research Institute, Anhui Academy of Agricultural Sciences, Hefei, Anhui, China

**Keywords:** soybean, cytoplasmic male sterility, uninucleate microspore, binucleate pollen, transcriptome, proteome

## Abstract

**Introduction:**

Heterosis is a critical phenomenon in crop improvement. Cytoplasmic male sterility (CMS) and Restorer gene (*Rf*) systems are essential components for heterosis-based breeding. However, the molecular mechanism underlying CMS remains largely unclear in soybean.

**Methods:**

We integrated a morphological investigation with comparative analyses of transcriptomic and proteomic changes in pollen from the CMS line W931A and its maintainer line, W931B, at the uninucleate microspore (UM) and binucleate pollen (BP) stages.

**Results:**

Compared to W931B, which had healthy, oval pollen grains, W931A showed shrunken or degraded pollen grains with an irregularly thickened endothelium and decreased starch accumulation. Transcriptomic comparisons revealed a total of 865 differentially expressed genes (DEGs) in W931A over the two stages. These genes were primarily associated with pentose and glucuronate interconversions, sphingolipid metabolism, and glycerolipid metabolism. Proteomic analysis revealed 343 differentially expressed proteins (DEPs), which were mainly involved in carbon metabolism, glycolysis/gluconeogenesis, and nitrogen metabolism. Consistently, Gene Ontology (GO) biological process terms related to pollen development were enriched among DEGs at the UM and BP stages. Notably, four genes with demonstrated roles in pollen development were differentially expressed, including AGAMOUS-LIKE 104, PROTEIN-TYROSINE-PHOSPHATASE 1, and PHOSPHOLIPASE A2. A total of 53 genes and the corresponding proteins were differentially expressed in W931A at both the UM and BP stages, and many of these were pectinesterases, polygalacturonases, peroxidases, and ATPases.

**Discussion:**

The results of this study suggest that pollen development in W931A is likely regulated through suppression of the identified DEGs and DEPs. These findings increase our understanding of the molecular mechanism underlying CMS in soybean, aiding future research into soybean fertility and promoting the efficient use of heterosis for soybean improvement.

## Introduction

Soybean is one of the most important oilseed crops in the world (Wang et al., 2021). Heterosis has yielded great economic benefits in grain crop breeding, including in maize, rice, and wheat (Chen and Liu, 2014; Gupta et al., 2019). The development of the cytoplasmic male sterility (CMS)/fertility restorer gene (*Rf*) system has been an important step in generating hybrid crop varieties. In rice, application of the CMS/*Rf* three-line system has significantly increased crop yield and improved production efficiency for hybrid seeds (Cheng et al., 2007; Chen and Liu, 2014). CMS is widely distributed among higher plants and typically results in a failure to produce viable pollen (Xiao et al., 2020). Studying CMS is an important way of gaining insights into interactions between the nucleus and the cytoplasm (Yang et al., 2022).

To elucidate the molecular mechanism underlying CMS, transcriptomic and proteomic approaches have been applied in many higher plants, such as *Brassica napus* (Yan et al., 2013; Ning et al., 2019), *Brassica campestris* (Chen et al., 2018), *Capsicum annuum* (Guo et al., 2017), *Gossypium hirsutum* (Suzuki et al., 2013), *Zea mays* (Zhang et al., 2020), and *Triticum aestivum* (Hao et al., 2021). Transcriptomic and proteomic analyses of *Brassica napus* Shaan2A anther mitochondria implied that the sterility gene may suppress expression of several nuclear transcription factors (TFs), affecting early anther development (Ning et al., 2019). Suzuki et al. (2013) conducted transcriptomic analysis of CMS and restorer lines, which showed that genes related to CMS were primarily involved in cell wall expansion. Anther mitochondrial proteins have also been identified and quantified in a three-line system; the results showed that the proteins involved were mainly related to fatty acid metabolism, amino acid metabolism, and protein processing pathways, indicating that energy deficiency hinders pollen development (Zhang et al., 2020).

CMS was first reported in soybean in 1985 (Davis, 1985). Li et al. (2015) analyzed flower buds in a soybean CMS line (NJCMS1A) and its maintainer line (NJCMS1B) *via* RNA-sequencing (RNA-seq), and concluded that male sterility may be related to dysfunction in some key genes and abnormalities in metabolic pathways. In the soybean CMS line, RNA-seq and small RNA-seq analyses were also performed to investigate the functional relevance of microRNAs (miRNAs) and their targets in regulating CMS (Zhang et al., 2021). It was found that miRNAs likely contribute to the CMS regulatory network by modulating the expression levels of genes involved in soybean CMS. Proteomic analysis was also completed for NJCMS1A and NJCMS1B using a strategy based on isobaric tags for relative and absolute quantification (iTRAQ), which revealed 15 differentially expressed proteins in the joint analysis (Li et al., 2016).

W931A is a soybean CMS line developed by crossing Zhongyou 89B and W206, followed by continuous backcrossing with W206 (Zhang and Dai, 1997). W206 is a recurrent parent that has been designated W931B. Cytological studies have revealed that W931A pollen abortion mostly occurs during the uninucleate microspore (UM) period, during which the nucleus and cytoplasm are degenerated and abnormal (Ren, 2005). However, many of the pollen grains (approximately 10%) contain round or ellipsoidal generative cells and may proceed to the binucleate pollen (BP) stage, although functional pollen cannot be generated (Ren, 2005). The molecular mechanisms regulating pollen development in W931A remain unknown, hindering its effective use in soybean improvement.

In the present study, we compared anther tissue transcriptomes and proteomes between W931A and W931B at the UM and BP stages. The aims of these analyses were to identify key regulators and metabolic pathways involved in CMS and to elucidate the underlying regulatory mechanisms. This comprehensive comparison provides novel insights into the molecular mechanism associated with soybean CMS.

## Materials and methods

### Plant materials

W931A and W931B were cultivated under consistent conditions in the test field at the Crop Research Institute, Anhui Academy of Agricultural Sciences (Hefei, Anhui, China). At the UM stage (when flower buds were 1.0-1.5 mm) and the BP stage (1.5-2.0 mm flower buds) (Li et al., 2005), anthers were collected and frozen in liquid nitrogen, then stored at -80°C prior to further experiments. There were three biological replicates per sample. W931B at the UM stage (W931B_UM) and at the BP stage (W931B_BP) were treated as the controls, whereas W931A at the UM stage (W931A_UM) and at the BP stage (W931A_BP) were considered the experimental samples.

### Observation *via* transmission electron microscopy (TEM)

Anthers from the BP and mature pollen (MP) stages were fixed in 2.5% (w/v) glutaraldehyde in 0.1 mol/L phosphate-buffered saline (PBS) at pH 7.4. After washing twice in PBS, samples were post-fixed in 1.0% OsO4, dehydrated in an ethanol series, and embedded in Epon812 resin as previously described (Fang et al., 2021). Embedded samples were cut into 2-µm-thick sections with an ultramicrotome (LEICA, Germany). Specimens were stained with uranyl acetate followed by lead citrate, then photographed under an H-7650 microscope (Hitachi, Japan) (Ning et al., 2018).

### RNA extraction, cDNA reverse transcription, and quantitative reverse transcription (qRT)−PCR analysis

Total RNA was isolated from three biological replicates of the W931A and W931B samples using RNAiso Plus reagent (TaKaRa, Japan), then treated with RNase-free DNase I (Promega, Madison, WI, USA). Subsequently, 0.5µg RNA per sample was used for first-strand cDNA synthesis using a HiScript II 1st Strand cDNA Synthesis kit (Vazyme, Nanjing, China). qRT−PCR was performed in technical triplicate on a LightCycler 96 (Roche, Switzerland) using the SYBR Green Premix Ex Taq™ II quantitative PCR system (TaKaRa, Japan). The amplification program consisted of a denaturation step at 95°C for 15 min; 40 cycles of 95°C for 10 s and 60°C for 20 s; and 72°C for 30 s. Gene expression was normalized using the 2^−ΔΔCT^ method (Livak and Schmittgen, 2001) using *TUBULIN1* as the internal reference gene. All primer sequences are listed in **Supplementary Table S1**.

### Transcriptomic analysis

RNA samples were sequenced on an Illumina HiSeq 2000 platform, generating 100-bp paired-end reads. The quality of each sample file was measured using FastQC v0.11.9. Illumina adapters, reads containing Ns (indicating that the corresponding bases could not be determined), and low-quality reads (those with Qphred values ≤ 20 for more than 50% of the total read length) were removed using Trim-galore v0.6.6, resulting in a set of clean reads. The reference genome file (Gmax_275_v2.0.fa) and the reference annotation file (Gmax_275_Wm82.a2.v1.gene.gtf) were downloaded from Phytozome v12. Clean reads were mapped to the soybean reference genome using Hisat2 v2.2.1. After mapping, reads were counted with HTSeq v0.13.5. Differential expression analysis was performed in the ‘DESeq2’ (v1.32.0) R package using the following screening criteria: |log2(Fold Change)| ≥ 1; *p* < 0.05; and false discovery rate (FDR) < 0.05. Gene ontology (GO) enrichment and Kyoto Encyclopedia of Genes and Genomes (KEGG) biochemical pathway enrichment analyses were performed on the DEGs using the ‘clusterProfiler’ (v4.0.5) package in R.

### Proteomic analysis

Total protein was extracted as previously described (Li et al., 2016) from three biological replicates of soybean samples at the UM and BP stages. Precipitated proteins were washed with ice-cold 90% ethanol containing 10 mM dithiothreitol. After drying at room temperature, precipitates were lysed in sodium dodecyl sulfate (SDS). Samples were centrifuged at 12,000 × g at room temperature for 10 min, then the supernatant was collected. The sample extraction procedure was repeated, and the resulting supernatant for each sample was combined with the supernatant from the first extraction to generate the total protein solution. Bicinchoninic acid reagent (Thermo Fisher Scientific, San Jose, CA, USA) was used to measure the protein content.

Based on the calculated protein content, 100 μg of total protein was removed from each sample and digested with Trypsin Gold (Promega, Madison, WI, USA) using a protein:trypsin ratio of 30:1. Samples were incubated at 37°C for 16 h. After trypsin digestion, peptides were labeled with iTRAQ tags following the manufacturer’s protocol for the 8-plex iTRAQ reagent (Applied Biosystems, Foster City, CA, USA). The labeled peptide mixtures were then pooled and pre-separated with strong cation exchange chromatography using the LC-20AB high-performance liquid chromatography (HPLC) pump system (Shimadzu, Kyoto, Japan). iTRAQ analysis was performed on a TripleTOF 5600 system (AB SCIEX, Concord, ON, Canada) combined with a Famos autosampler (LC Packings) and an LC20-AD Nano HPLC instrument (Shimadzu) as previously reported (Du et al., 2014). Proteome Discoverer v1.2.0.339 (Thermo Fisher Scientific) was used to transform the raw data into MGF files. Protein identification and quantitation were performed in Mascot v2.3.0 (Matrix Science, London, UK) to compare the iTRAQ data against data in Soybase (https://soybase.org/). Differentially expressed proteins (DEPs) were classified as those with *p* < 0.05 in a least significant difference test, FDR < 0.05, and > 1.5-fold change between W931A and W931B.

### Interaction analysis

DEP interaction analysis was conducted using the STRING database (https://string-db.org/). Both known and predicted protein-protein interactions were included. The option “Multiple sequences” was selected to analyze interactions among 53 differentially expressed soybean proteins of interest. Protein sequences were downloaded from Soybase.

## Results

### Morphological differences between W931A and W931B anthers

**Figure 1:**
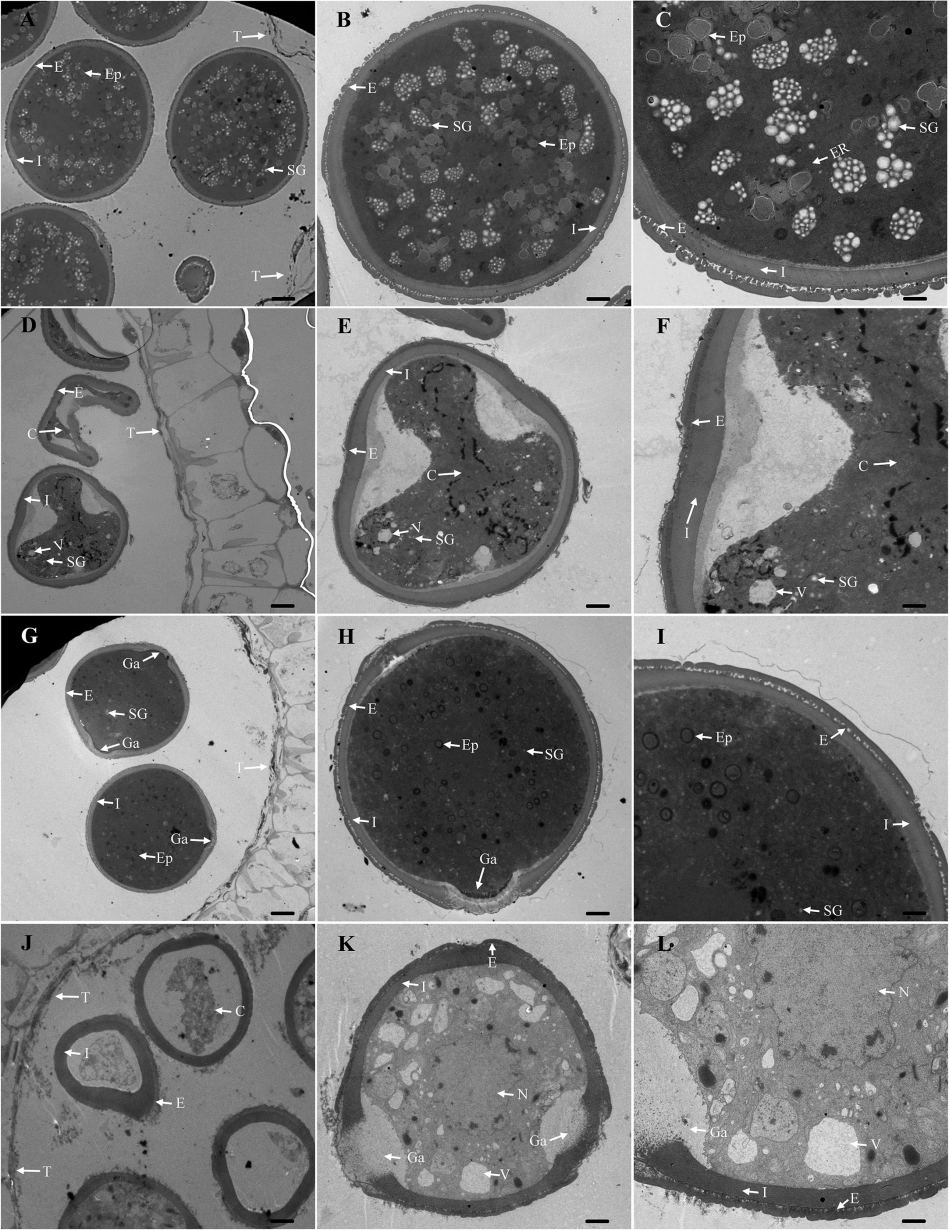
Transmission electron microscopy (TEM) images of anthers from the cytoplasmic male sterility (CMS) line W931A and its maintainer line, W931B. **(A–C)**: Binucleate pollen (BP) grain from the maintainer line W931B at the BP stage. **(D–F):** BP grain from the male sterile line W931A at the BP stage. **(G–I)**: Mature pollen grain from the maintainer line W931B. **(J–L)**: Mature pollen grain from the male sterile line W931A. C, content; E, exine; Ep, elaioplast; ER, endoplasmic reticulum; Ga, germinal aperture; I, intine; N, nucleolus; SG, starch granule; T, tapetum; V, vacuole. Scale bars = 10 μm **(A, D, G, J)**, 5 μm **(B, E, H, K)**, and 2 μm **(C, F, I, L)**.

W931A is an excellent male sterile line with a strong stem that produces more than twice as many floral buds as regular fertile varieties (Ren, 2005). To determine the cytological basis of functional deficiencies at the BP stage, we compared the ultramicroscopic structures of developing anthers and pollen grains between W931A and its maintainer line, W931B, using TEM. The results showed that the anthers of W931A and W931B developed similarly; for example, the tapetal layers of both lines were degraded at the BP stage (**Figures 1A, D**).

At the BP stage, W931B pollen grains were filled with condensed cytoplasm. Starch granules and lipid materials were extensively accumulated, and the protectum was smooth (**Figures 1A–C**). In contrast, W931A pollen grains showed an irregular shape and abnormal development (e.g., few starch granules, large vacuoles, irregularly thickened intine, and uneven protectum) (**Figures 1D–F**). At the MP stage, W931B pollen grains were round and exhibited a concentrated cytoplasm, in which a number of elaioplasts and abundant lipid compounds could be observed (**Figures 1G–I**). Comparatively, W931A pollen grains showed severe degeneration and vacuolization of the cytoplasm (**Figures 1J–L**).

### Comparative transcriptomic analysis

To reveal the molecular mechanism underlying CMS, we analyzed the transcriptome and the proteome of developing W931A and W931B pollen grains at the UM and BP stages. In total, 12 cDNA libraries were constructed and sequenced (three biological replicates of two genotypes at two stages). Clean reads were mapped to the Wm82 reference genome, and approximately 468 million reads were aligned, with an average mapping rate of 86.48% per sample. The average GC content was 44.18% (**Supplementary Table S2**).

The transcriptomes were then compared between W931A and W931B pollen grains at the UM and BP stages. A total of 2,259 differentially expressed genes (DEGs) were identified at the UM stage, including 598 genes that were up-regulated and 1,661 that were down-regulated in W931A (**Supplementary Figure S1A**, **Table S3**). In contrast, a total of 1,962 DEGs were identified at the BP stage, including 1,063 up-regulated and 899 down-regulated genes (**Supplementary Figure S1B**, **Table S4**). The expression levels of 15 randomly-selected genes were assessed using qRT-PCR to validate the sequencing results. The coefficient of determination (R^2^) for the two data types was near 1 (R^2^ = 0.9304), indicating that the RNA-seq data were robust and suitable for further analysis (**Supplementary Figure S2**).

To gain insight into the functions of the DEGs, GO term enrichment (p < 0.05) was assessed, including GO biological process (GBP), cellular component (CC), and molecular function (MF) terms. At the UM stage, GO analysis of the 2,259 DEGs revealed significant enrichment of the GBP terms “pectin catabolic process”, “photosynthesis, light harvesting in photosystem I”, “cell wall modification”, “regulation of pH”, and “response to light stimulus” (**Supplementary Table S5**). At the BP stage, the 1,962 DEGs were significantly enriched in the GBP terms “pectin catabolic process”, “photosynthesis, light harvesting in photosystem I”, “cell wall modification”, “response to light stimulus”, and “protein-chromophore linkage” (**Supplementary Table S6**). KEGG biochemical pathway enrichment (*p* < 0.05) was also investigated, and revealed enrichment of pathways including “pentose and glucuronate interconversions”, “photosynthesis-antenna proteins”, “metabolic pathways”, “plant-pathogen interaction”, and “ribosome biogenesis in eukaryotes” at the UM stage (**Supplementary Table S8**), and “metabolic pathways”, “biosynthesis of secondary metabolites”, “flavonoid biosynthesis”, “photosynthesis-antenna proteins”, and “tyrosine metabolism” at the BP stage (**Supplementary Table S9**).

**Figure 2:**
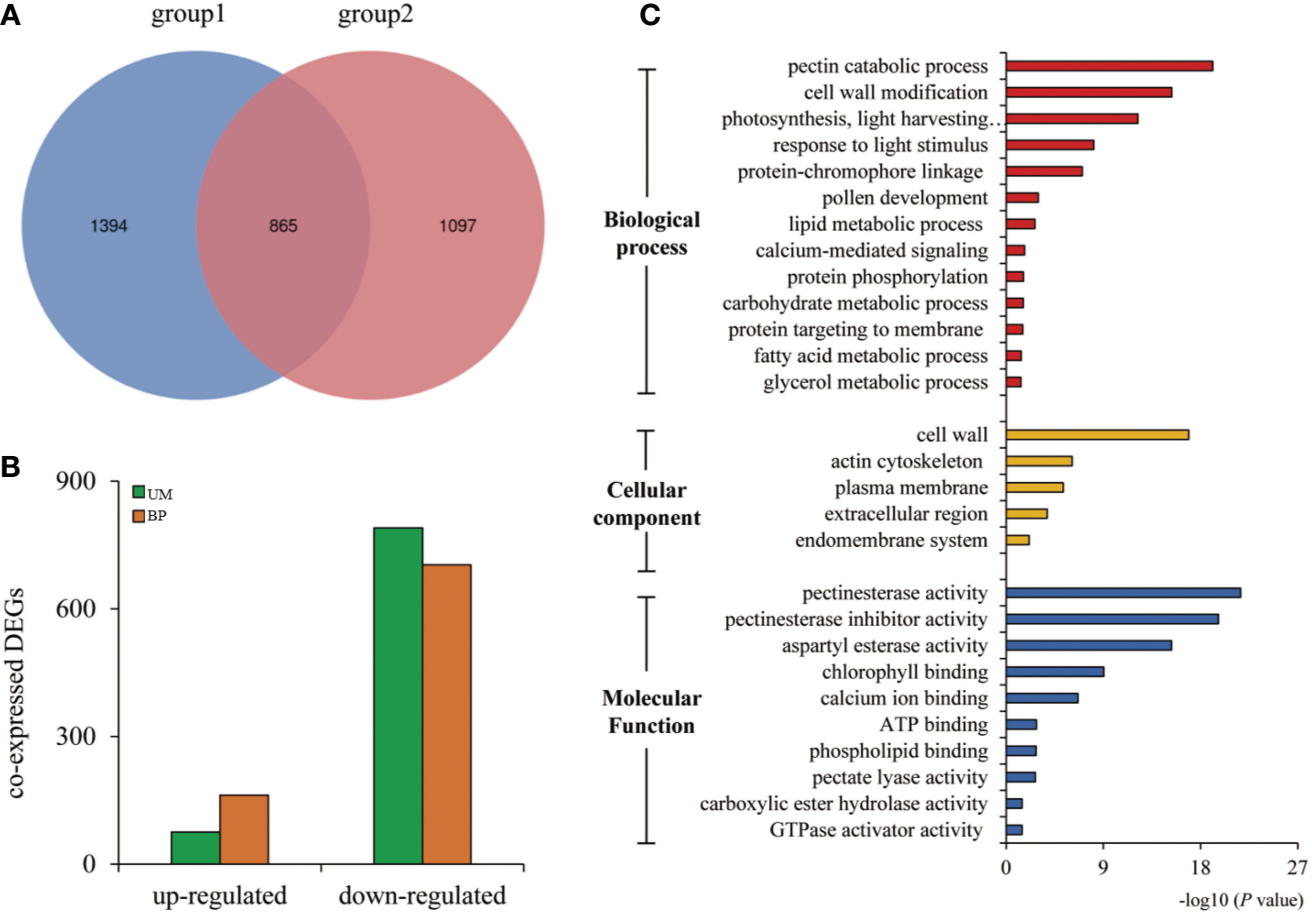
Analysis of differentially expressed genes (DEGs) in W931A at the uninucleate microspore (UM) and binucleate pollen (BP) stages. **(A)** Venn diagram showing the number of unique and overlapping DEGs at the UM stage (group1) and the BP stage (group2). **(B)** Number of up-regulated and down-regulated genes at the UM and BP stages, respectively. **(C)** Most highly enriched Gene Ontology (GO) terms among the DEGs.

**Figure 3:**
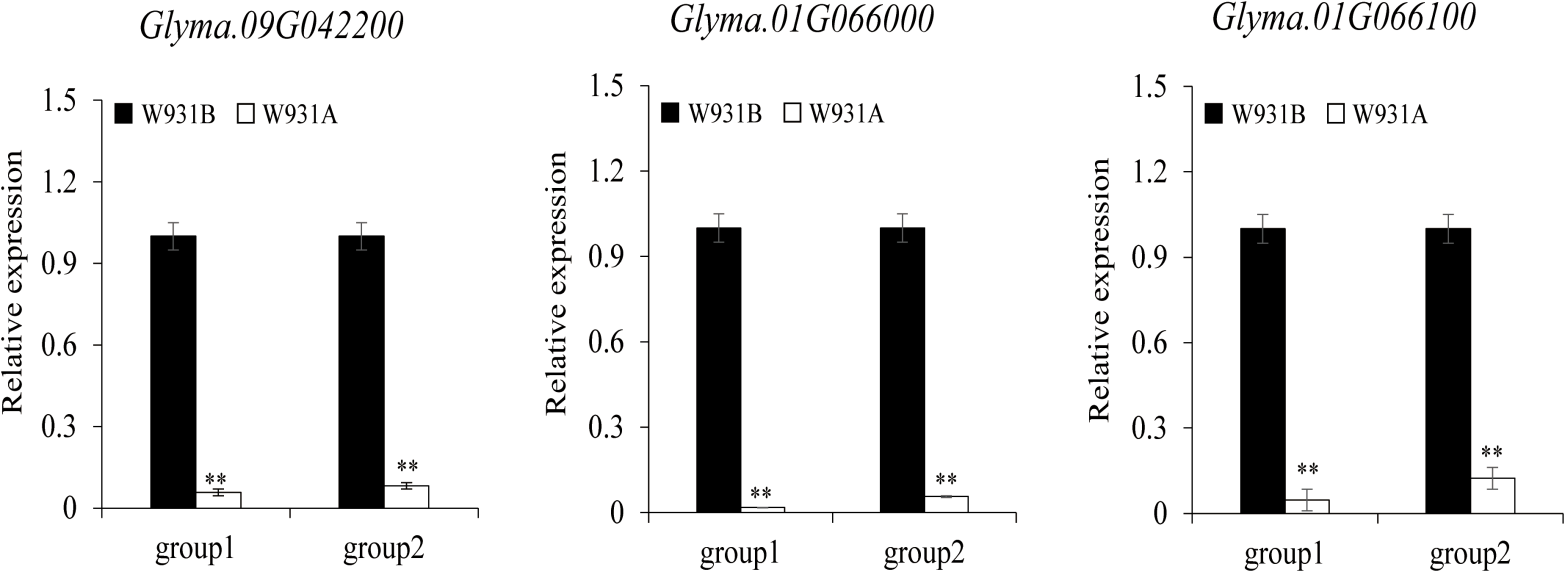
Expression levels of candidate genes with the “pectinesterase” molecular function annotation as determined with quantitative reverse transcription (qRT)-PCR. Relative expression levels are shown for the uninucleate microspore (UM) stage (group1) and for the binucleate pollen (BP) stage (group2).

Further analysis showed that a total of 865 DEGs were shared between the two developmental stages (**Figure 2A**). We also found that most of the DEGs were down-regulated; at the UM stage, there were 10-fold more down-regulated than up-regulated genes; at the BP stage, there were approximately five-fold more down-regulated than up-regulated genes (**Figure 2B**). To determine the putative functions of the shared DEGs, we conducted a GO enrichment analysis. The 865 DEGs were primarily associated with the GBP terms “pectin catabolic process”, “cell wall modification”, “photosynthesis, light harvesting in photosystem I”, “response to light stimulus”, “protein-chromophore linkage”, and, notably, “pollen development” (**Supplementary Table S7**). The most significantly enriched MF terms were “pectinesterase activity” and “pectinesterase inhibitor activity” (**Figure 2C**, **Supplementary Table S7**). Many of the shared DEGs that had those MF annotations encoded pectinesterases (PMEs)/PME inhibitors and pectate lyases. For example, *Rfk1* encodes a PME/PME inhibitor protein that is involved in pectin metabolism and regulates fertility restoration in wheat (Chen et al., 2021). *Glyma.09G042200* encoded a PME/PME inhibitor enzyme that was strongly down-regulated in W931A at both the UM and BP stages. qRT-PCR results also showed marked down-regulation of three PME/PME inhibitor genes in W931A compared to W931B (**Figure 3**).

In W931A, 1,394 and 1,097 DEGs were uniquely expressed at the UM and BP stages, respectively (**Supplementary Tables S3, S4**). GO term analysis of the 1,394 unique DEGs at the UM stage revealed enrichment of the GBP terms “mitochondrion organization”, “hydrogen peroxide catabolic process”, “positive regulation of mitochondrial translation”, “response to oxidative stress”, and “cell wall modification” (**Supplementary Table S5**), whereas the 1,097 DEGs unique to the BP stage were significantly enriched in the GBP terms “auxin-activated signaling pathway”, “defense response to other organism”, “flavonoid biosynthetic process”, “fatty acid biosynthetic process”, and “brassinosteroid metabolic process” (**Supplementary Table S6**). These data indicated that many genes expressed in the pollen had unique spatiotemporal expression patterns.

### Comparative proteomic analysis

**Figure 4:**
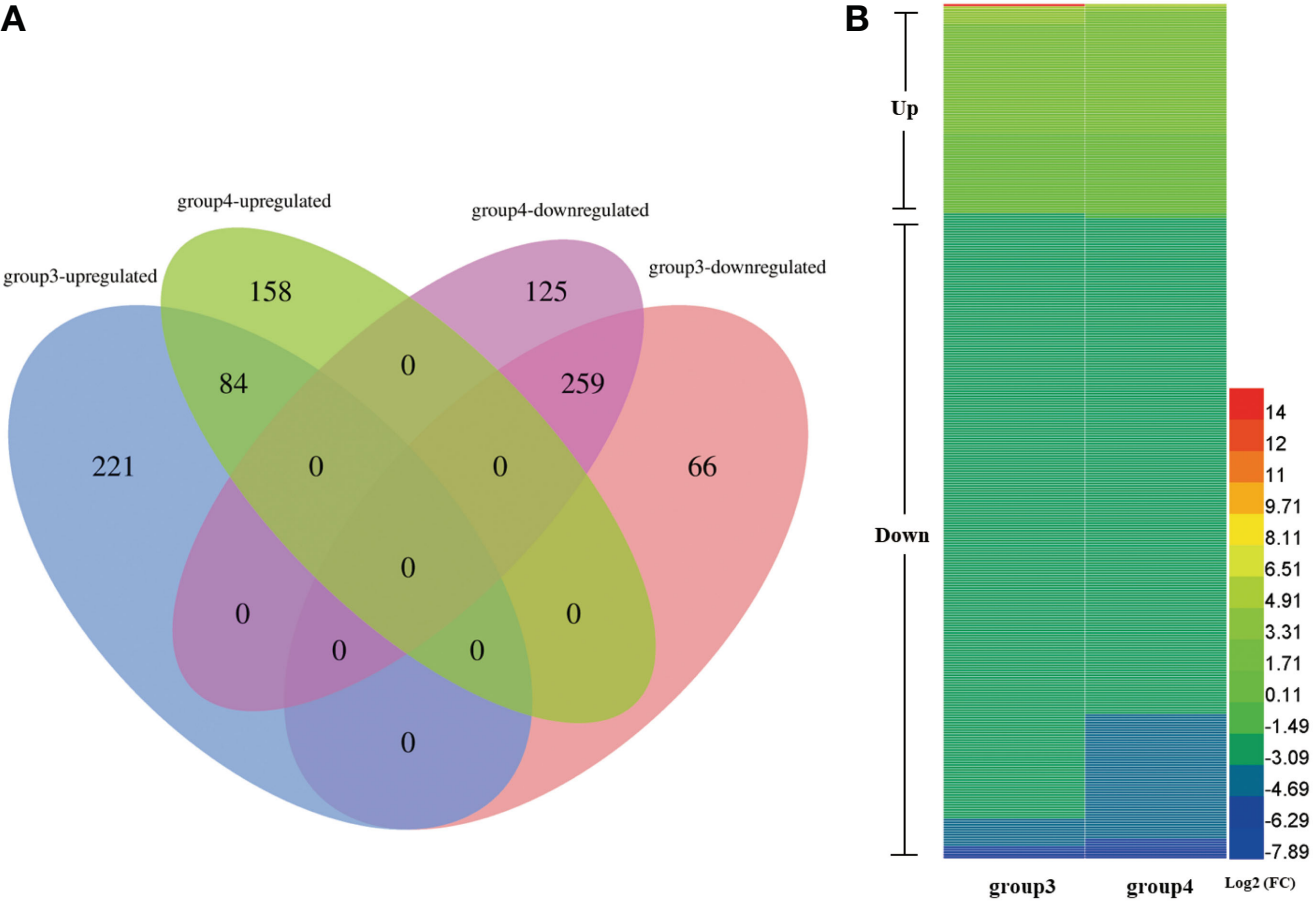
Expression patterns of differentially expressed proteins (DEPs) in W931A at the uninucleate microspore (UM) and binucleate pollen (BP) stages. **(A)** Venn diagram showing unique and overlapping DEPs in W931A at the UM and BP stages (group3 and group4, respectively). **(B)** There were 343 DEPs shared between the UM and BP stages.

We next carried out a proteomic analysis to identify factors involved in soybean CMS at the protein level. To analyze DEPs between W931A and W931B, proteins expressed in the anthers of W931A and W931B plants at the UM and BP stage were analyzed using iTRAQ technology. A total of 630 DEPs were identified in W931A compared to W931B at the UM stage, with 305 up-regulated and 325 down-regulated (**Figure 4A**; **Supplementary Table S11**). At the BP stage, 242 proteins were up-regulated in W931A compared to W931B, whereas 384 were down-regulated (**Figure 4A**; **Supplementary Table S12**).

GO term enrichment analysis revealed that the GBP terms “glycolytic process”, “one-carbon metabolic process”, “carbohydrate metabolic process”, “response to toxic substance”, and “cell wall modification” were significantly enriched in W931A at the UM stage (**Supplementary Table S14**). At the BP stage, markedly enriched GBP terms included “glycolytic process”, “glucose metabolic process”, “one-carbon metabolic process”, “pectin catabolic process”, and “cell wall modification” (**Supplementary Table S15**). However, several key molecular functions were also enriched at the UM stage, such as “nuclear import signal receptor activity”, “glutathione transferase activity” and “NAD binding”, indicating that enzymes with these functions tended to be more active during UM development (**Supplementary Table S14**). In contrast, GO terms that were specifically enriched at the BP stage included “electron carrier activity”, “structural constituent of ribosome”, “mRNA binding”, and “phosphatidic acid binding” (**Supplementary Table S15**), indicating that proteins associated with these MF terms played important roles at the BP stage.

Further analysis revealed 343 DEPs in common between the two plant developmental stages (**Figure 4B**; **Supplementary Table S13**). Enriched GO terms in the shared DEP set included “glycolytic process”, “cell wall modification”, “pectin catabolic process”, “glucose metabolic process”, and “carbohydrate metabolic process” (**Supplementary Table S16**). The largest category of enriched MF GO terms was “pectinesterase activity” (**Supplementary Table S16**). The shared DEPs were mapped to a total of 31 KEGG pathways (**Supplementary Table S18**). The most significantly enriched pathway was “metabolic pathways”, followed by “carbon metabolism” and “glycolysis/gluconeogenesis”. The primary physiological function of carbohydrate metabolism in an organism is to provide carbon sources. Some proteins related to monosaccharide and polysaccharide metabolic pathways were also evidently expressed; proteins with the pathway annotations “galactose metabolism”, “fructose and mannose metabolism”, and “starch and sucrose metabolism” were enriched in W931A (**Supplementary Table S18**).

### DEG and DEP correlation analysis

To identify whether there was general concordance between mRNA and associated protein levels during pollen development, the transcriptomic and proteomic data were compared by conducting a correlation analysis between the DEGs and DEPs. One hundred and forty-four common-DEGs and DEPs were expressed at the UM stage (**Supplementary Tables S3, S11**). Seventy-eight common-DEGs and DEPs were expressed at the BP stage (**Supplementary Tables S4, S12**). The expression profiles of 53 sets of DEGs and associated DEPs found at both the UM and BP stages are shown in **Supplementary Figure S3** and **Table 1**. Further analysis of the 53 shared DEGs with similar corresponding protein expression showed that they primarily encoded PMEs/PME inhibitors, polygalacturonases (PGs), peroxidases, ATPases, and sugar transport proteins.

**Table 1:** The expression profiles of 53 common-DEGs and DEPs at both the UM and BP stages.

ID	log_2_FC(group1)	log_2_FC(group2)	Log_2_FC(group3)	Log_2_FC(group4)
Glyma.09G042200	-13.71	-3.66	-2.00	-2.77
Glyma.09G042100	-11.87	-2.63	-2.31	-3.78
Glyma.01G066000	-11.56	-3.05	-2.00	-2.45
Glyma.01G066100	-10.23	-2.42	-2.81	-3.89
Glyma.17G172700	-9.88	-3.05	-2.48	-3.20
Glyma.01G027000	-8.49	-1.74	-1.82	-2.07
Glyma.01G145900	-8.42	-2.50	-2.48	-3.39
Glyma.09G035100	-8.34	-1.75	-2.50	-3.36
Glyma.04G143200	-8.29	-3.00	-3.01	-4.53
Glyma.10G198100	-8.23	-2.57	-2.36	-2.99
Glyma.02G037800	-8.03	-2.56	-1.71	-1.97
Glyma.02G080000	-7.99	-3.22	-1.62	-2.20
Glyma.14G006100	-7.85	-2.15	-2.38	-3.16
Glyma.14G074000	-7.82	-2.57	-1.73	-2.11
Glyma.07G003000	-7.80	-1.63	-2.35	-2.89
Glyma.16G154000	-6.98	-1.24	-2.38	-2.25
Glyma.16G083700	-6.98	-3.15	-2.60	-4.08
Glyma.19G070900	-6.94	-3.06	-1.92	-2.22
Glyma.06G207300	-6.89	-2.53	-2.38	-3.41
Glyma.09G261800	-6.57	-2.26	-3.10	-3.43
Glyma.05G206500	-6.42	-2.07	-1.78	-1.87
Glyma.10G235500	-6.18	-1.43	-1.93	-2.04
Glyma.04G022800	-6.05	-2.60	-2.60	-3.12
Glyma.09G252700	-5.78	3.04	-4.69	-4.23
Glyma.10G249400	-5.62	-1.28	-3.34	-5.54
Glyma.15G082300	-5.45	-1.96	-1.99	-2.41
Glyma.18G063400	-5.32	-1.84	-2.53	-2.81
Glyma.13G230300	-5.03	-1.54	-2.38	-3.36
Glyma.03G133600	-4.99	-1.52	-2.75	-2.99
Glyma.07G226000	-4.45	-1.46	-2.35	-3.48
Glyma.10G054500	-4.22	-1.39	-2.07	-2.81
Glyma.10G229600	-3.96	-1.84	-2.62	-3.34
Glyma.18G240000	-3.94	3.80	-4.86	-4.44
Glyma.13G141600	-3.62	-1.11	-2.04	-2.28
Glyma.05G163300	-3.54	-1.06	-2.22	-2.27
Glyma.13G050000	-3.23	5.14	-3.01	-2.93
Glyma.09G284200	-3.19	5.34	-3.32	-4.06
Glyma.15G004300	-2.86	1.94	-1.95	-2.23
Glyma.19G079400	-2.79	-2.10	-1.72	-1.89
Glyma.08G162100	-2.61	-2.09	-1.88	-2.06
Glyma.08G068100	-2.46	2.35	-2.46	-3.12
Glyma.03G238800	-2.37	-1.76	-1.77	-2.33
Glyma.15G265500	-2.15	-2.48	-2.07	-2.35
Glyma.05G090100	-2.14	2.00	-1.95	-3.86
Glyma.07G181700	-2.14	1.95	-3.16	-3.89
Glyma.05G178300	-1.97	-1.39	-1.61	-1.80
Glyma.20G081400	-1.94	5.57	-3.01	-3.16
Glyma.04G172800	-1.32	3.20	-2.51	-3.41
Glyma.08G082900	1.53	-2.12	1.82	1.67
Glyma.11G247600	1.81	1.49	3.89	1.93
Glyma.04G123800	2.52	3.39	2.46	2.08
Glyma.06G193500	3.49	2.37	3.07	1.83
Glyma.03G003900	4.29	4.89	-1.88	-2.39

### GO and KEGG enrichment analysis of DEGs and DEPs

**Figure 5:**
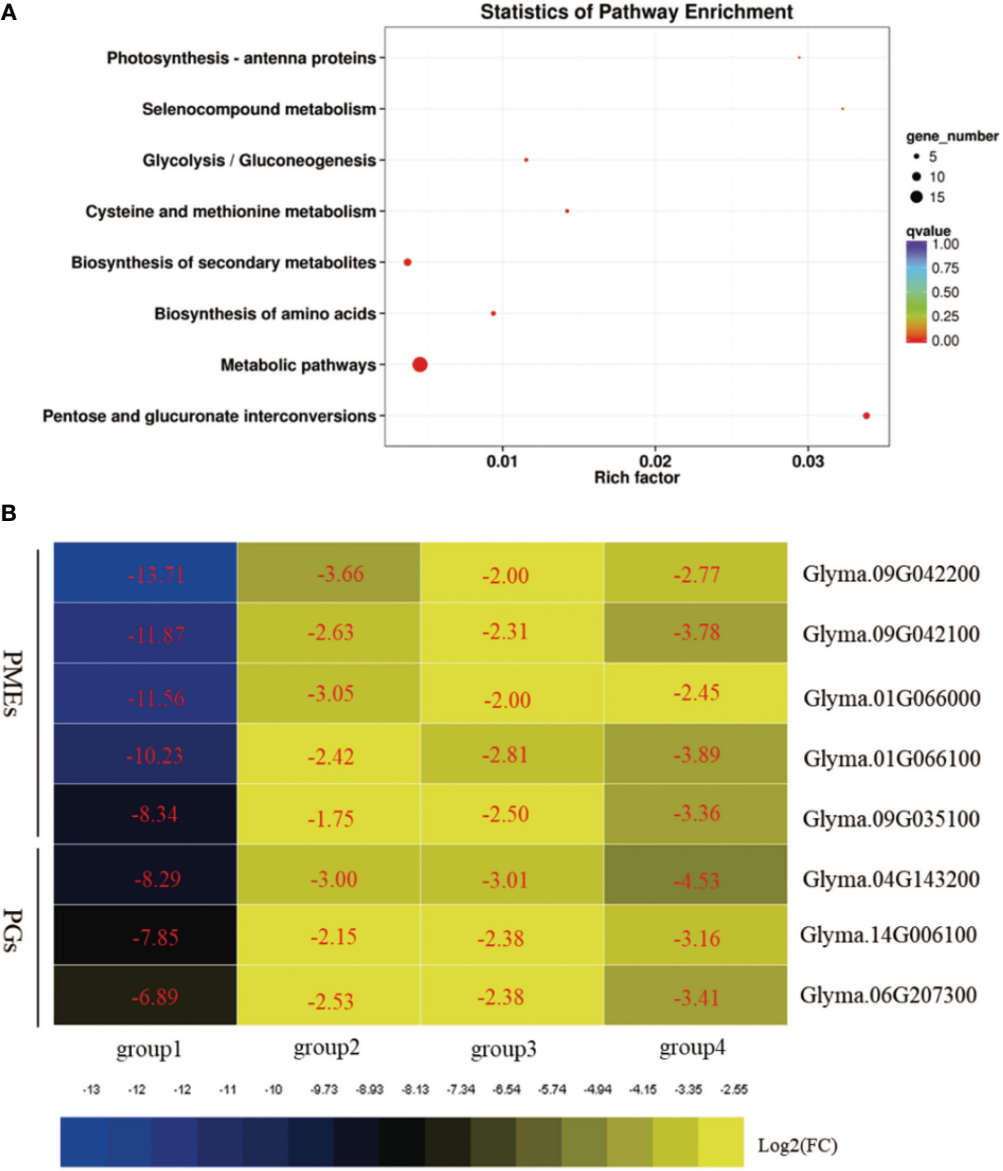
Biological pathway analysis of differentially expressed genes (DEGs) and differentially expressed genes (DEPs) shared between the uninucleate microspore (UM) and binucleate pollen (BP) stages. **(A)** Enriched Kyoto Encyclopedia of Genes and Genomes (KEGG) biochemical pathway terms among the shared DEGs and DEPs. **(B)** Heatmap illustrating the expression levels of selected pectinesterases (PMEs) and polygalacturonases (PGs) involved in cell wall and pollen tube development. Group1, DEGs in W931A compared to W931B at the UM stage; group2, DEGs in W931A at the BP stage; group3, DEPs in W931A at the UM stage; group4, DEPs in W931A at the BP stage.

A total set of 53 DEGs/DEPs that were differentially expressed at both the UM and BP stages were identified as genes/proteins that affect biological processes during soybean pollen development. GO term analysis of this set showed marked enrichment of the GBP terms “cell wall modification”, “pectin catabolic process”, “carbohydrate metabolic process”, “hydrogen peroxide catabolic process”, and “response to oxidative stress” (**Supplementary Table S19**). The MF terms “pectinesterase activity”, “carboxylic ester hydrolase activity”, “polygalacturonase activity”, “carbohydrate binding”, and “peroxidase activity” were also enriched (**Supplementary Table S19**). Analysis of the transcriptomic and proteomic data demonstrated consistent activation of PMEs/PME inhibitors at the mRNA and protein levels, indicating the importance of these proteins in regulating soybean fertility. In addition, the KEGG pathways “pentose and glucuronate interconversions”, “metabolic pathways”, “biosynthesis of amino acids”, “biosynthesis of secondary metabolites”, and “glycolysis/gluconeogenesis” were significantly enriched (**Figure 5A**; **Supplementary Table S20**).

### Metabolic pathways in pollen development

Pollen development is an intensely energy-consuming process (Hanson and Bentolila, 2004; Linke and Börner, 2005; Geng et al., 2018). The process of carbon fixation is primarily responsible for generating usable carbon for plants (Saxena et al., 2020). Here, transcriptomic analysis revealed 30 DEGs in pollen that participated in pentose and glucuronate interconversions (**Supplementary Table S10**) proteomic analysis showed that there were 26 DEPs associated with carbon metabolism, 19 related to glycolysis/gluconeogenesis, 10 related to pentose and glucuronate interconversions, six associated with galactose metabolism, six related to fructose and mannose metabolism, eight associated with starch and sucrose metabolism, and four related to the pentose phosphate pathway (**Supplementary Table S18**).

We also found that five DEPs were involved in the tricarboxylic acid (TCA) cycle, with three of these proteins up-regulated in W931A and two down-regulated (**Supplementary Table S17**). The two down-regulated DEPs associated with the TCA cycle were phosphoenolpyruvate carboxykinase (ATP) proteins. We also found that seven DEPs were associated with oxidative phosphorylation, with five up-regulated and two down-regulated in W931A compared to W931B (**Supplementary Table S17**). The two down-regulated proteins were components of ATPase 9.

### Putative DEGs and DEPs related to pollen development

MADS-box genes encode TFs that have important biological functions in the formation of male flower organs (Linke et al., 2003). In *Arabidopsis thaliana*, *AGL104* (*At1g22130*) is expressed specifically in the pollen (Adamczyk and Fernandez, 2009). Two agamous-like MADS-box (*AGL*) genes with the GBP “pollen development” were markedly expressed at both the UM and BP stages (**Supplementary Table S7**). *Glyma.13G086400* (log_2_(FC) = -5.83) and *Glyma.14G168700* (log_2_(FC) = -5.99) were clearly down-regulated in W931A at the UM stage (**Supplementary Table S3**) and similarly down-regulated at the BP stage (log_2_(FC) = -2.09 and -2.48, respectively) (**Supplementary Table S4**). *AGL104* expression levels changed during anther abortion, suggesting that *AGL104* may have an important molecular function in pollen production. Moreover, the *Arabidopsis* gene *tyrosine-phosphatase 1 (PTEN1)* encodes a pollen-specific phosphatase that is essential for pollen development (Gupta et al., 2002). Phospholipase A2 (PLA2) plays critical roles in *Arabidopsis* pollen development, germination, and tube growth (Kim et al., 2011). In the present study, two genes with the GBP “pollen development”, *Glyma.08G241500* (*PTEN1*) and *Glyma.07G127900* (*PLA2-like*), were also down-regulated at both the UM and BP stages (**Supplementary Tables S3, S4**). They were significantly expressed at the transcriptional level, but not at the protein level, indicating that a complex post-transcriptional regulatory network may exist in W931A to regulate male sterility.

Pectin is one of the most important polysaccharides for plant cell wall growth (Zhang et al., 2020). We found that pectin catabolic processes occurred at both the transcriptional and protein levels. In W931A at the UM stage, 39 DEGs (two up-regulated and 37 down-regulated) had annotations associated with pectin catabolic processes (**Supplementary Table S5**). Similarly, there were 34 DEGs (seven up-regulated and 27 down-regulated) in W931A at the BP stage encoded pectate lyases or PMEs (**Supplementary Table S6**), and seven DEPs (all down-regulated) at the UM stage (**Supplementary Table S14**), and 10 DEPs (all down-regulated) at the BP stage that were pectate lyases or PMEs (**Supplementary Table S15**). Five PMEs were also identified in the set of 53 shared DEGs and DEPs; these were down-regulated at both the mRNA and protein levels (**Figure 5B**). Furthermore, among the 53 candidate DEGs were three DEGs encoding PGs (*Glyma.06G207300*, *Glyma.14G006100*, and *Glyma.04G143200*), three DEGs encoding peroxidases (*Glyma.02G037800*, *Glyma.01G027000*, and *Glyma.10G198100*), one DEG encoding tubulin alpha-3 (*Glyma.16G154000*), and one DEG encoding ATPase 9 (*Glyma.15G004300*) (**Table 1**).

### Predicted interactions between candidate genes

**Figure 6:**
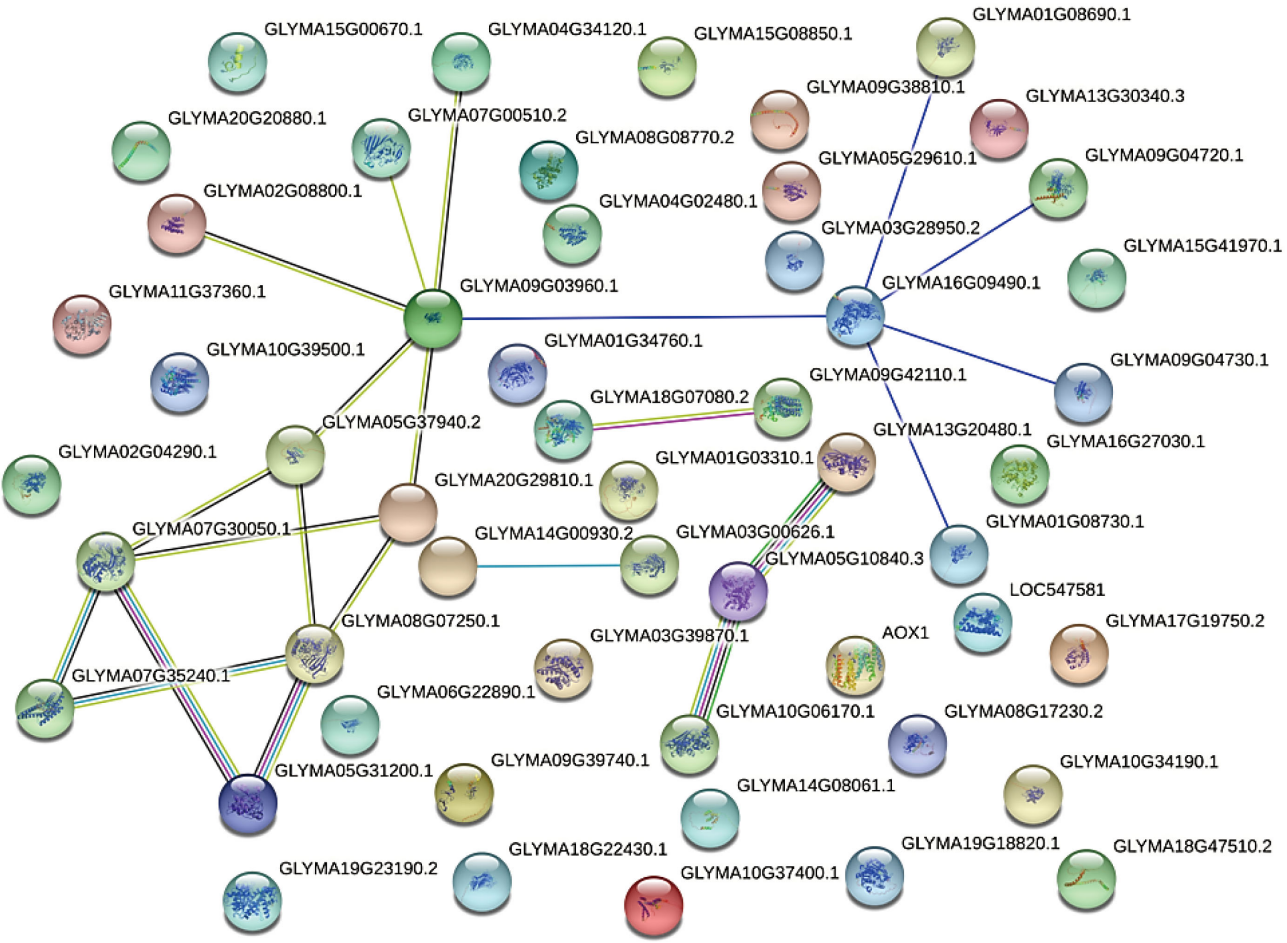
Predicted interaction network for 53 proteins that may be involved in cytoplasmic male sterility. Line color indicates the type of interaction evidence. Bright blue, known interaction (curated database); pink, known interaction (experimentally determined); green, predicted interaction (gene neighborhood); red, predicted interaction (gene fusion); dark blue, predicted interaction (gene co-occurrence); green, potential interaction (text mining); black, potential interaction (co-expression); light blue, potential interaction (protein homology).

Interactions between the candidate proteins corresponding to the 53 shared DEGs and DEPs were next predicted using STRING 10.0. The results indicated that these proteins may form a complex network regulating pollen development in W931 CMS (**Figure 6**). The putative protein regulatory network included PME, β-galactosidase, actin-depolymerizing factor, and S-adenosylmethionine synthetases as core proteins involved in pollen development (**Figure 6**). There was a potential interaction mechanism between four PME proteins (PME28, PME58, and two PME1s) and β-galactosidase 15 (BGAL 15). The PMEs and BGAL 15 may be involved in the cell wall loosening associated with pollen expansion during microspore development. In okra (*Abelmoschus esculentus*), *AeADF1* has been shown to promote pollen germination and pollen tube growth (Dong et al., 2021). Here, actin depolymerizing factor 7 (GmADF7) and its interacting proteins were significantly down-regulated in W931A, indicating that they may inhibit pollen germination and thus cause soybean sterility.

## Discussion

CMS is a maternally-inherited trait that prevents pollination due to pollen abortion (Hanson, 1991). Understanding the entire process and the molecular mechanisms involved in pollen development are the bases for exploration of male sterility and will allow it to be used in soybean improvement. CMS is known to occur in more than 200 plant species and has been used extensively in crop hybrid breeding (Li et al., 2017), including in soybean, the most abundantly cultivated oilseed crop. However, heterosis has not yet been universally applied in soybean breeding, partially because the mechanism controlling CMS is unclear. In the present study, we analyzed the molecular mechanism underlying soybean CMS through comprehensive transcriptomic and proteomic analyses at the two major stages that are critical for pollen development (Mascarenhas, 1990). We revealed marked changes in the expression of genes and proteins involved in pollen development and cell wall modification (**Supplementary Tables S7, S16, S19**). Genes that are expressed early in pollen development primarily encode cytoskeletal proteins and proteins involved in cell wall synthesis or starch accumulation, whereas genes that are expressed late during pollen development are considered necessary for pollen maturation or pollen tube growth (Guyon et al., 2000). Supporting the results of our analyses, studies in most species have confirmed that a large amount of the RNA required for pollen tube growth accumulates in pollen grains prior to germination (Guyon et al., 2000).

PMEs regulate the mechanical and chemical properties of cell walls *via* methyl esterification of cell wall pectin (Li et al., 2002; Jiang et al., 2005; Hepler et al., 2013). PMEs play important roles in plant cell wall synthesis and metabolism; abnormal cell wall development affects pollen vitality and can lead to male sterility (Chen et al., 2021). In the wheat line CMS-K, abnormal changes in the pollen intine wall structure and the ATPase activity response are likely important in pollen grain abortion (Yao et al., 2002; Chen et al., 2021). In Chinese cabbage-pak-choi (*Brassica rapa ssp. chinensis, syn. B. campestris ssp. chinensis*), *BcMF3* encoding a PME. Transgenic Arabidopsis expressing *BcMF3* produce some deformed pollen grains and exhibit the abnormal phenomenon of pollen tube rupture during pollen germination (Liu et al., 2006). The PME-encoding genes *Glyma.09G042200*, *Glyma.09G042100, Glyma.09G035100, Glyma.01G066100*, and *Glyma.01G066000* (**Table 1**) were markedly down-regulated in W931A at both the mRNA and protein levels, indicating that these genes play important roles in pollen development. In addition to PMEs, other DEGs that had functions associated with cell wall biosynthesis and regulation and that were expressed specifically in the pollen may also have been involved in pollen development (Becker et al., 2003; Shi et al., 2015), such as the gene encoding a PG (Hocq et al., 2020; Ye et al., 2020; Wu et al., 2022). PG is a cell wall hydrolysis and loosening enzyme that plays a role in pollen maturation and pollen tube growth by degrading pectin. The PG gene family is specifically expressed in the pollen of *Arabidopsis*, maize (Honys and Twell, 2003; Lu et al., 2021), and Chinese cabbage (Wang et al., 2005). Three PGs, Glyma.06G207300, Glyma.14G006100, and Glyma.04G143200, were significantly down-regulated at both the mRNA and protein levels (**Figure 5B**), suggesting that they may have important roles in regulating soybean fertility.

Pollen specifically expressed tubulin genes have been demonstrated in some species, such as *Oryza sativa*, *Picea wilsonii*, *Populus tremuloides*, and *Arabidopsis thaliana* (Cheng et al., 2001; Yoshikawa et al., 2003; Oakley et al., 2007; Yu et al., 2009). Of the six α-tubulin genes in *Arabidopsis*, one (*TUA1*) is preferentially expressed in pollen (Kopczak et al., 1992). Eight α-tubulin genes are expressed in poplar, but *PtTUA6* and *PtTUA8* are only abundantly expressed in pollen (Oakley et al., 2007). In soybean, *Glyma.16G154000* is predicted to encode a TUA3 protein and has the GBP annotations “microtubule-based process”, “mitotic cell cycle”, and “microtubule cytoskeleton organization” (**Supplementary Table S19**). In the CMS line W931A, TUA3 was down-regulated at both the mRNA and protein levels (**Table 1**), indicating that it may affect pollen development by regulating microtubule formation. Such genes identified in this study require further analysis to determine their roles and functions in soybean CMS.

An insufficient energy supply can damage male gamete development, leading to male sterility (Chen and Liu, 2014). Compared with normal lines, CMS lines have a lower level of energy metabolism (Teixeira et al., 2005), and genes related to energy metabolism directly contribute to the recovery of fertility in plant CMS lines (Liu et al., 2018). At the metabolic level, W931A showed decreased glycolysis/gluconeogenesis, carbon metabolism, starch and sucrose metabolism, and TCA cycle activity during microspore development (**Supplementary Tables S17, S18**). A collection of such abnormalities in the energy metabolism system can lead to a shortage of energy formation and supply in the anthers, thus affecting microsporogenesis and pollen development (Chen and Liu, 2014).

## Conclusions

We here performed transcriptomic and proteomic analyses to understand the molecular mechanisms regulating CMS in soybean. Soybean pollen abortion in W931A was likely regulated by multiple pathways involved in pollen development, pectin catabolism, responses to oxidative stress, carbon metabolism, the TCA cycle, and oxidative phosphorylation. We established a number of DEGs and DEPs that may be involved in these processes, such as PGs and PMEs. Our results provide novel insights and candidate genes for further studies into the mechanism underlying CMS in soybean; this will ultimately promote the application of W931A in soybean heterosis and breeding.

## Data availability statement

The original contributions presented in the study are publicly available. This data can be found here: NCBI, PRJNA895173.

## Author contributions

ZH and JL conceived the concept of the review. DW, YW, LZ, YY, QW, GH, and WW compiled the literature. DW and YW designed the figures. DW and YW wrote the paper. All authors contributed to the article and approved the submitted version.
