# Supplementary material for: Integrated transcriptomic and proteomic analysis of a cytoplasmic male sterility line and associated maintainer line in soybean
Source: Front Plant Sci. 2023 Feb 2;14:1098125. doi: 10.3389/fpls.2023.1098125 (PMC9933710; doi:10.3389/fpls.2023.1098125)
Supplement: Supplementary file 1 [file DataSheet_1.zip › Supplementary materials/Supplementary materials.docx]

**Supplementary Table S1**. Primers were used in this study

| Name | Forward Primers | Reverse Primers |
| --- | --- | --- |
| Tubulin1 | GGAGTTCACAGAGGCAGAG | CACTTACGCATCACATAGCA |
| Glyma.03g003900 | ATCTAATCCTTGCCCCGCTG | TCAAGTCCACCACGTGCTTT |
| Glyma.04g143200 | CAACGGAAGTGCAGCAACAG | AATGGGGATGGCTTGCTTCT |
| Glyma.04g172800 | GATGCTGTGAATCGTCCAAATT | CCGTGAATCTGTAGACCATGTA |
| Glyma.05g090100 | TAAACTTGTCGTGTCTACCTCC | GATGATCAGAACCCTTCAATGC |
| Glyma.05g206500 | TAGGGGATCCCACGGAAAGT | GCGCTTTCACAAGGTCCAAG |
| Glyma.07g181700 | CCTATTCCAGACACCAATGACT | GCTACATTGTGAGCATAAGAGC |
| Glyma.08g068100 | CCGTGTTCTGATTCACACAAAT | GACTTCAGTGCTTCAACATCTG |
| Glyma.08g082900 | CACTCACCACTTCAAAACACTC | GATGAGAGAGCCATGGAAGAAG |
| Glyma.11g247600 | CATATTTGACGCCAAGGCCG | CCAAGATCCGCCTCAACTGT |
| Glyma.09g035100 | AGGGTCCTTATTCTAGGGTGAT | GAACAACACGATTATACCCACG |
| Glyma.18g240000 | GGCAGTGAAGAAGAACATGAAC | GACATATCGATCGATCCTCTCC |
| Glyma.09g252700 | CAATAGAGCTGCAGACAATGTC | GAGTTCATGTTCTGCTTCACTG |
| Glyma.20g081400 | AGGCAGAACATTTTGTCCAATC | CTGTTGTGTTAGAAGCTGCATT |
| Glyma.09g042200 | ATGGACGGGTTCCAAGACAC | AGCCAATTCGGGTTCTCCAG |
| Glyma.01g066000 | TTTGGTGTTCATGTGCACTATG | CCCATTTTCATGAATTTACAGGGAC |
| Glyma.01g066100 | GGCATTGCGTATTTCGGGTG | CATTGTCGCCAAGCGTTCTT |

**Supplementary Table S2**. The transcriptome sequencing dates of W931A and W931B

| Sample | Replicate | Total reads | GC % | Total Mapped | Multiple mapped | Uniquely mapped |
| --- | --- | --- | --- | --- | --- | --- |
| W931A_UM | A_UM1 | 36876362 | 44.5 | 31215331(84.64%) | 1865831(5.05%) | 29349500(79.58%) |
|  | A_UM2 | 47707058 | 44.55 | 41927551(87.88%) | 4486652(9.40%) | 37440899(78.48%) |
|  | A_UM3 | 42571010 | 44.76 | 36761786(86.35%) | 1630070(3.82%) | 35131716(82.52%) |
| W931A_BP | A_BP1 | 37700116 | 44.18 | 31593571(83.80%) | 1931067(5.12%) | 29662504(78.68%) |
|  | A_BP2 | 38543162 | 44.48 | 31674482(82.17%) | 1531634(3.97%) | 30142848(78.20%) |
|  | A_BP3 | 39314818 | 44.37 | 33304862(84.71%) | 2580043(6.56%) | 30724819(78.15%) |
| W931B_UM | B_UM1 | 42239552 | 44.47 | 38428196(90.97%) | 1576110(3.73%) | 36852086(87.24%) |
|  | B_UM2 | 38094320 | 45.94 | 31462569(82.59%) | 4086306(10.72%) | 27376263(71.86%) |
|  | B_UM3 | 35842164 | 44.53 | 31353189(87.47%) | 1568266(4.37%) | 29784923(83.10%) |
| W931B_BP | B_BP1 | 37488146 | 46.75 | 33565730(89.53%) | 11457277(30.56%) | 22108453(58.97%) |
|  | B_BP2 | 40971426 | 44.18 | 36256751(88.49%) | 2637716(6.43%) | 33619035(82.05%) |
|  | B_BP3 | 31534372 | 44.32 | 28118011(89.16%) | 2993819(9.49%) | 25124192(79.67%) |


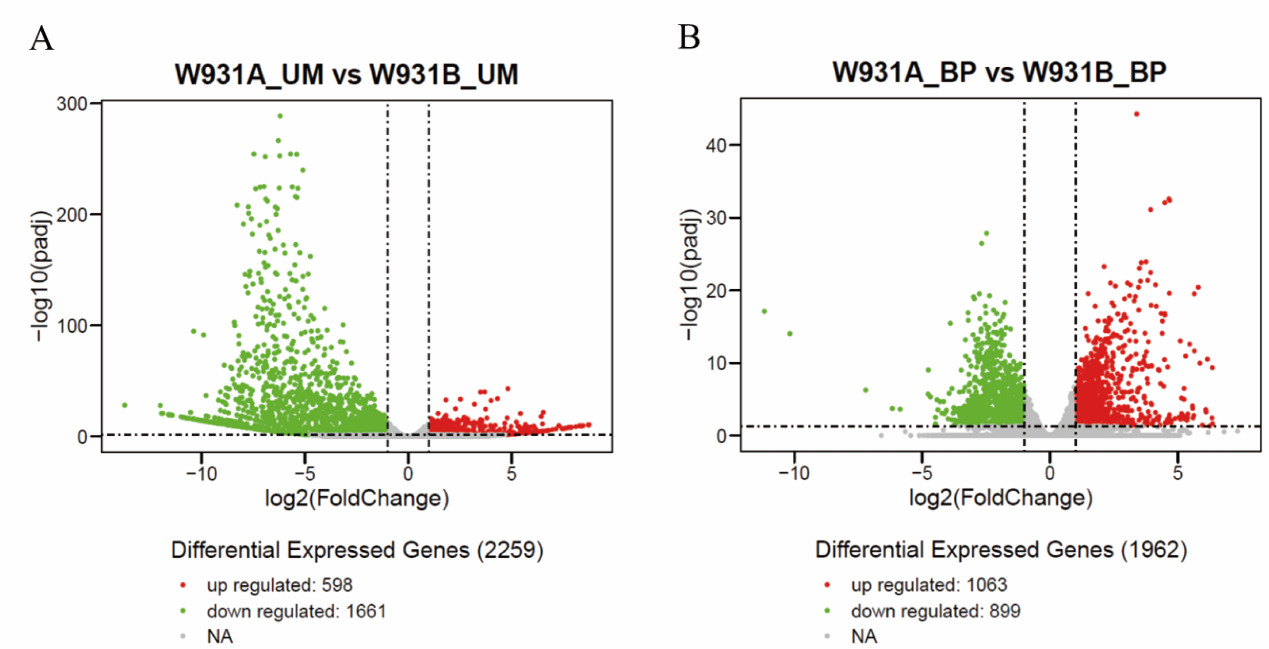


**Supplementary Figure S1.** Differential gene expression patterns of W931A in comparison with W931B at UM and BP stages. (**A**) DEGs in W931A at the UM stage; (**B**) DEGs in W931A at the BP stage. The differences of comparison are reflected in the volcano map. Grey represents non-significantly differential genes, while red and green represent upregulation and downregulation genes, respectively; the x-axis denotes log_2_ (Fold Change), and the y-axis denotes log_10_ (*P* value).


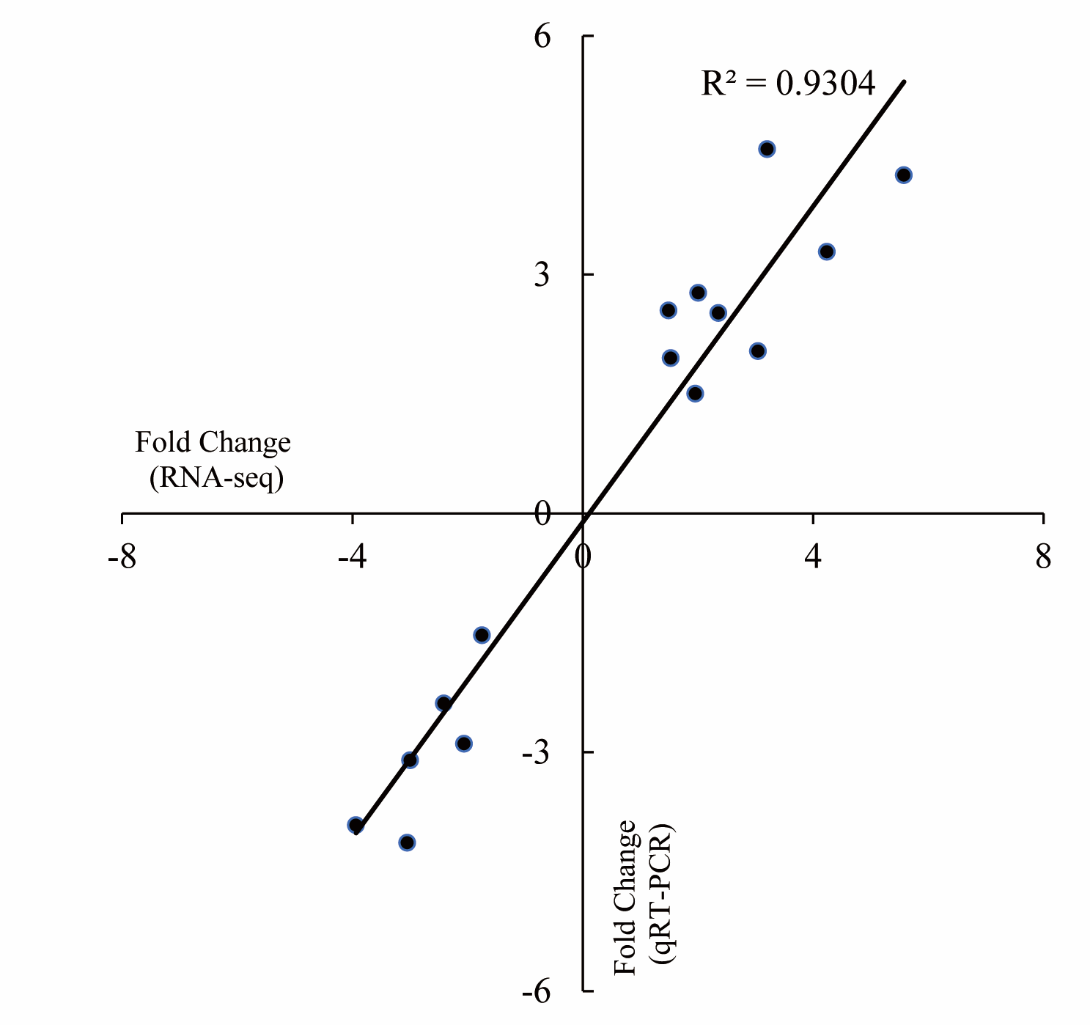


**Supplementary Figure S2.** Correlation analysis between RNA-seq (x-axis) and qRT-PCR results (y-axis). 15 DEGs were selected for qRT-PCR analysis to determine the accuracy and repeatability of RNA-seq.


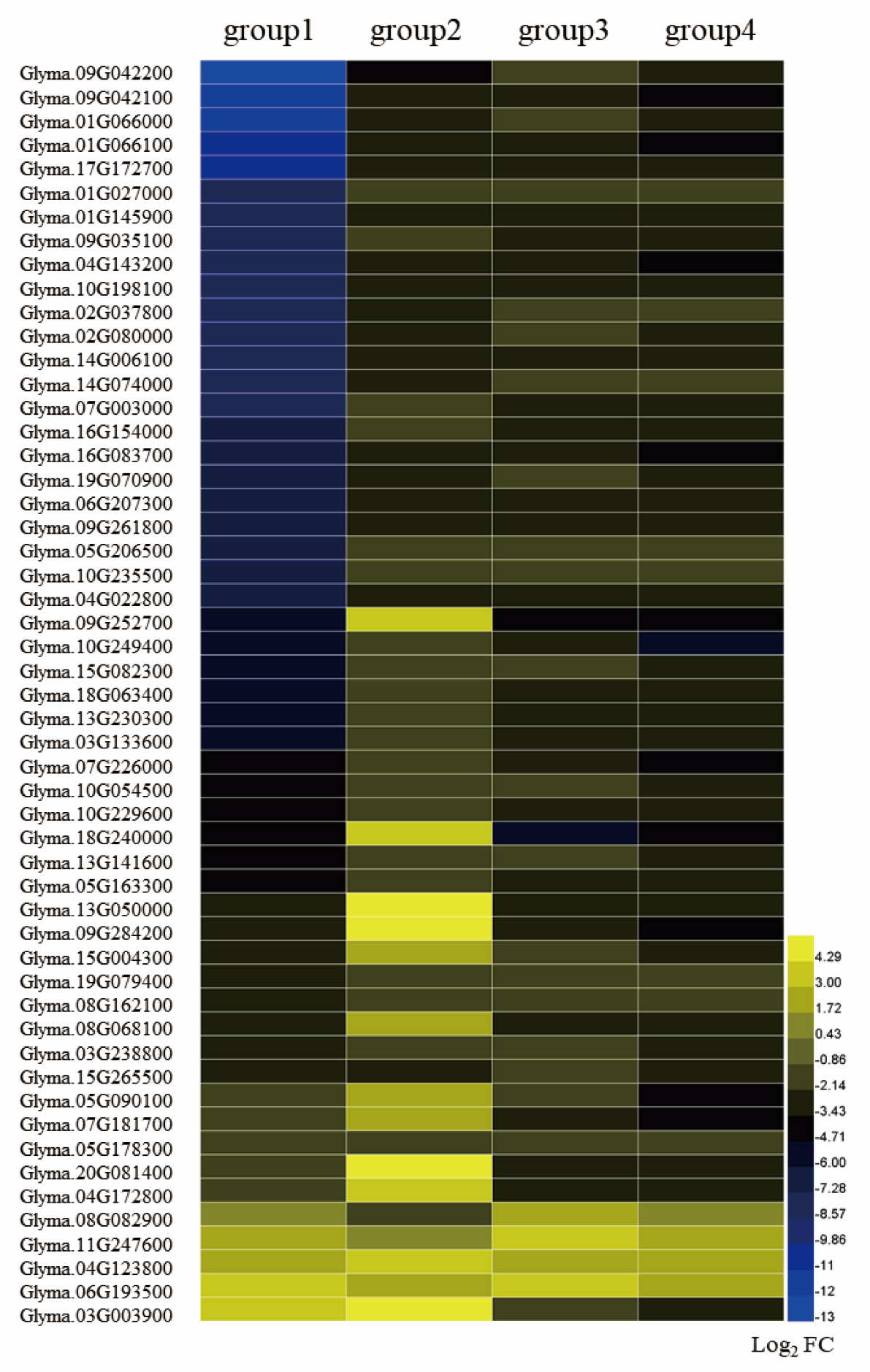


**Supplementary Figure S3**. The expression levels of 53 common-DEGs and DEPs at both the UM and BP stages.
